# Psilocybin Therapy of Psychiatric Disorders Is Not Hampered by hERG Potassium Channel–Mediated Cardiotoxicity

**DOI:** 10.1093/ijnp/pyab085

**Published:** 2021-12-06

**Authors:** Benjamin Hackl, Hannes Todt, Helmut Kubista, Karlheinz Hilber, Xaver Koenig

**Affiliations:** Department of Neurophysiology and -Pharmacology, Center for Physiology and Pharmacology, Medical University of Vienna, Vienna, Austria

## Abstract

Psilocybin, a hallucinogen contained in “magic” mushrooms, holds great promise for the treatment of various psychiatric disorders, and early clinical trials are encouraging. Adverse cardiac events after intake of high doses of psilocybin and a trial reporting QT interval prolongation in the electrocardiogram attributed to the drug’s main metabolite, psilocin, gave rise to safety concerns. Here we show that clinical concentrations of psilocin do not cause significant human ether-a-go-go-related gene (hERG) potassium channel inhibition, a major risk factor for adverse cardiac events. We conclude that hERG channel blockage by psilocin is not liable for psilocybin- associated cardiotoxic effects.

Psilocybin (4-phosphoryloxy-N,N-dimethyltryptamine) is an indole alkaloid found in a family of mushrooms commonly named “magic mushrooms” and well known for its hallucinogenic effects in humans. After ingestion, psilocybin is immediately metabolized to psilocin in the body. This metabolite is crucial for the alkaloid’s psychotropic properties (Madsen et al., 2021).

Scientific interest in psilocybin has dramatically increased within the last decade. In 2020, 13 clinical trials were ongoing that tested psilocybin for use in various psychiatric conditions (Tullis, 2021). Completed early clinical studies have indicated great therapeutic potential of psilocybin in treating anxiety, depression, end-of-life distress, and various forms of addiction (e.g., Davis et al., 2021). Recently, the FDA has given psilocybin a “breakthrough therapy designation” for treating major depressive disorder and treatment-resistant depression. Thus, psilocybin is on the edge of making a significant impact on therapies on hand for psychiatric medicine.

Successful drug development will require a thorough evaluation of psilocybin’s safety profile when administered to humans. Potential cardiotoxic effects of psilocybin have not yet been sufficiently studied. Concerning are reports of severe cardiac arrhythmias, cardiac arrest, contractile dysfunction, and myocardial infarction after intake of psilocybin at high doses (Nef et al., 2009; Li et al., 2019). Psilocybin-induced abnormalities in electrocardiogram (ECG) parameters [ST-elevation (Nef et al., 2009), QT interval prolongation (Li et al., 2019)] suggest that the alkaloid may disturb cardiac ion channel activity. Not psilocybin itself but its metabolite psilocin may be at least partly responsible for the cardiotoxicity observed after psilocybin intake. Thus, in rats, application of psilocin produced ECG abnormalities, aberrant intraventricular conduction, and arrhythmias (Borowiak et al., 2006). A recently performed phase I clinical trial on 12 healthy adult human participants revealed that psilocin prolongs the QTc interval in the ECG (Dahmane et al., 2021). These authors concluded that, at a clinical psilocybin dose of 25 mg, there is a limited potential of QTc prolongation. Confirming this view, psilocybin did not significantly alter QTc intervals at a dose of 25 mg compared with placebo in another study in 23 healthy participants (Becker et al., 2021). Higher drug doses (42–59 mg) have the potential to prolong QTc above the threshold of regulatory concern (Dahmane et al., 2021).

Drug-induced QT prolongation is known to be associated with an increased risk of life-threatening torsade de pointes arrhythmias (Redfern et al., 2003). The most common reason for drug-induced QT prolongation is blockage of ether-a-go-go-related gene (ERG) potassium channels, which are crucial determinants of the repolarization phase of the cardiac action potential. Cardiotoxicity due to ERG channel blockage is also a common reason for drug failure in preclinical safety trials (Redfern et al., 2003). Considerable affinity of psilocybin for human ERG (hERG) channels is unlikely because its charged phosphate group (Figure 1A) impedes plasma membrane diffusion and thereby prevents the drug from reaching the channel’s canonical binding site located within the cytoplasmic inner vestibule. Psilocin, on the other hand, is uncharged (Figure 1A) and may thus reach the binding site. We therefore reasoned that psilocin may block hERG channels in clinically relevant concentrations, which could induce QT interval prolongation.

**Figure 1.**
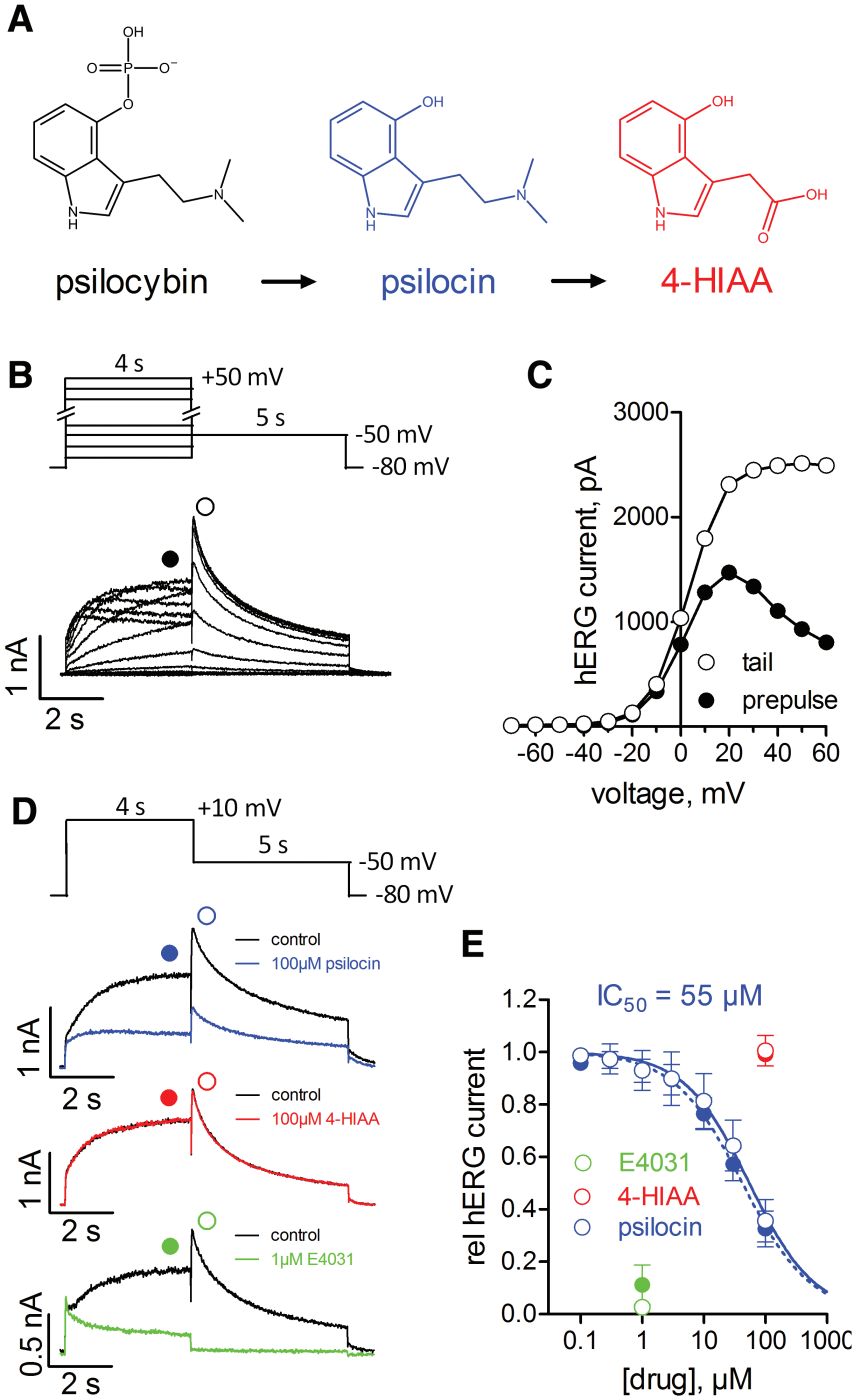
Supraclinical concentrations of psilocin inhibit hERG channels heterologously expressed in tsA-201 cells. (A) Chemical structures of psilocybin (black) and its metabolites psilocin (blue) and 4-hydroxyindole-3-acetic acid (4-HIAA, red). (B) Potassium currents through hERG channels in tsA-201 cells recorded with the whole-cell patch clamp technique as previously described (Koenig et al., 2013). Voltage-clamp pulse protocol (top; pulses applied every 15 seconds) with representative original current traces. (C) Current-voltage relationship of the experiment displayed in B; throughout the figure, filled or open circles represent evaluation of current amplitude at the end of the 4-second prepulse (+10 mV) or at the peak of tail current (−50 mV), respectively. (D) Voltage-clamp protocol (applied every 15 seconds) to test for the effect of drugs on hERG channels and respective representative potassium currents before (control) and after equilibration with different concentrations of psilocin (Serobac, PSI-410-FB-10; 100 mM stock dissolved in DMSO, final concentrations: 0.1–100 µM), 4-HIAA (PubChem 7061393; AKos Consulting & Solutions AKOS003270455; 100 mM stock in DMSO, final concentration: 100 µM), and E4031 (selective hERG channel inhibitor; Sigma Aldrich M5060; 1 mM stock in H_2_O, final concentration: 1 µM). (E) Concentration response curve for hERG current inhibition by psilocin (blue); fit of the data to a Hill equation yielded an IC_50_ value of 55 ± 10 µM (42 ± 6 µM) with a Hill coefficient of 0.8 ± 0.1 (0.8 ± 0.1) at −50 mV (+10 mV). n = 5 independent experiments; data are expressed as means ± SD. In addition, relative hERG current level on application of 100 µM 4-HIAA (n = 6, red) or 1 µM E4031 (n = 5, green) is displayed.

For testing this hypothesis, hERG channels were heterologously expressed in tsA-201 cells, and inhibition of currents through these channels by psilocin was investigated with the whole-cell patch clamp technique. Typical currents through hERG channels and their current-voltage relationship are displayed in Figure 1, B and C, respectively. Psilocin reduced hERG currents in a concentration-dependent manner. The concentration required to generate 50% inhibition (IC_50_) was 55 µM (Figure 1D,E). The psilocin metabolite 4-hydroxyindole-3-acetic acid (4-HIAA), applied at a 100 µM concentration, did not affect hERG currents. The average peak plasma concentration of the non-glucuronidated psychoactive psilocin is 20 ng/mL or 0.1 µM after administration of a typical psilocin dose for humans of 25 mg (Brown et al., 2017; Becker et al., 2021; Dahmane et al., 2021; Davis et al., 2021; Kolaczynska et al., 2021). Hence, a 500-fold higher psilocin concentration than that actually reached in human plasma was needed to cause relevant hERG channel inhibition. Even a 100-fold margin between the free maximal plasma concentration and hERG IC_50_ ensures a high degree of safety for drugs prescribed to psychiatric patients (Redfern et al., 2003). This implies that hERG blockade by psilocin does not account for psilocybin-associated cardiotoxic effects. Further, hERG blockade also cannot explain the previously published QT interval–prolonging effect of psilocin in a phase I clinical trial (Dahmane et al., 2021). Here, however, it should be mentioned that low in vitro hERG affinity does not entirely rule out association of a drug with torsade de pointes arrhythmias. For example, amiodarone (1400-fold margin) and diphenhydramine (880-fold margin) are torsadogenic and among the rare exceptions that have been described (Redfern et al., 2003).

Finally, we envision that, in the course of mushroom poisoning, as in the recently reported case of a 68-year-old woman (Li et al., 2019), hERG channel blockage by supraclinical concentrations of psilocin may contribute to QT prolongation and arrhythmia generation, especially in the presence of other risk factors. Among those are a prolonged baseline QT interval, ion channel mutations, bradycardia, abnormal electrolyte levels, preexisting cardiovascular disease, drug–drug interactions, and genetic variants influencing drug metabolism (Redfern et al., 2003).

Taken together, these data allow us to conclude that hERG channel blockage by psilocin in concentrations reached in clinical settings is not liable for psilocybin-associated cardiotoxic effects. 
